# Classification of age groups and task conditions provides additional evidence for differences in electrophysiological correlates of inhibitory control across the lifespan

**DOI:** 10.1186/s40708-023-00190-y

**Published:** 2023-05-08

**Authors:** Christian Goelz, Eva-Maria Reuter, Stephanie Fröhlich, Julian Rudisch, Ben Godde, Solveig Vieluf, Claudia Voelcker-Rehage

**Affiliations:** 1 Institute of Sports Medicine, Paderborn University, Paderborn, Germany; 2 Department of Sport and Health Sciences, Technical University of Munich, Munich, Germany; 3 Department of Neuromotor Behavior and Exercise, Institute of Sport and Exercise Sciences, University of Münster, Wilhelm-Schickard-Str. 8, 48149 Münster, Germany; 4 School of Business, Social and Decision Sciences, Constructor University, Bremen, Germany; 5 Division of Epilepsy and Clinical Neurophysiology, Department of Neurology, Boston Children’s Hospital, Harvard Medical School, Boston, MA USA

**Keywords:** Machine learning, Decoding, EEG/ERP, Flanker, Selective attention, Development

## Abstract

The aim of this study was to extend previous findings on selective attention over a lifetime using machine learning procedures. By decoding group membership and stimulus type, we aimed to study differences in the neural representation of inhibitory control across age groups at a single-trial level. We re-analyzed data from 211 subjects from six age groups between 8 and 83 years of age. Based on single-trial EEG recordings during a flanker task, we used support vector machines to predict the age group as well as to determine the presented stimulus type (i.e., congruent, or incongruent stimulus). The classification of group membership was highly above chance level (accuracy: 55%, chance level: 17%). Early EEG responses were found to play an important role, and a grouped pattern of classification performance emerged corresponding to age structure. There was a clear cluster of individuals after retirement, i.e., misclassifications mostly occurred within this cluster. The stimulus type could be classified above chance level in ~ 95% of subjects. We identified time windows relevant for classification performance that are discussed in the context of early visual attention and conflict processing. In children and older adults, a high variability and latency of these time windows were found. We were able to demonstrate differences in neuronal dynamics at the level of individual trials. Our analysis was sensitive to mapping gross changes, e.g., at retirement age, and to differentiating components of visual attention across age groups, adding value for the diagnosis of cognitive status across the lifespan. Overall, the results highlight the use of machine learning in the study of brain activity over a lifetime.

**Graphical Abstract:**

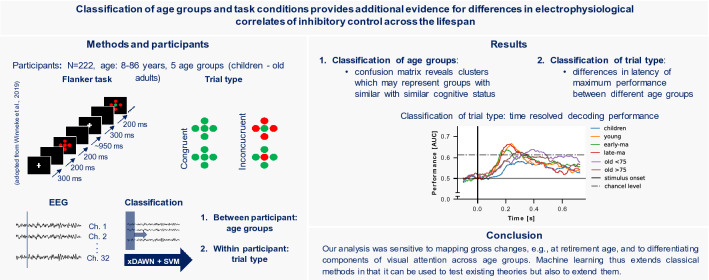

## Introduction

Selective attention (as part of inhibitory control) describes the ability to focus on relevant information while simultaneously suppressing irrelevant or distracting input which is essential for the accomplishment of complex tasks [1]. This ability changes throughout the lifespan. Whereas selective attention develops in children due to the differentiation of brain areas and networks, the opposite is noticeable in older adults, namely, a reduction in selective attention likely related to dedifferentiation processes in the brain [2, 3]. Focusing on the neuronal response to sensory stimuli measured with electroencephalography (EEG) differences in the distribution, amplitude, and latency of event-related potentials (ERP) were reported for different age groups [3]. Comparing six different age groups Reuter et al. [4] confirmed a u-shaped function of ERP markers for encoding and processing speed (i.e., P1, N1, N2, and P3 latencies), markers of visual processing and attention (i.e., P1 and N1 amplitudes) as well as gradual changes in markers of cognitive processing (N2, P3 amplitudes, and P3 distribution). Moreover, results suggest that different neural mechanisms underly performance in children and older adults [4]. The u-shaped function in previous ERP findings suggests that ERP components are similar between children and older adults despite fundamentally different mechanisms, i.e., differentiation in children versus dedifferentiation in older adults. It is unclear whether these differences are reflected in distinctive brain activation patterns and to what extent lifelong changes in electrophysiological markers can be detected at the level of individual trials.

Recently, the use of machine learning techniques to study experimental effects in EEG studies gained popularity as a complement to classical ERP analyses. These methods are referred to as multivariate pattern analysis (MVPA) or decoding approaches and are based on classification algorithms developed in the field of brain–computer interfaces (BCI) [5]. The main idea is to train a machine learning model based on single-trial EEG data that allows to classify a certain behavior or experimental condition. This involves the automatic detection of generalizable multivariate patterns associated with the behavior or experimental condition. Targeting the information content on a single-trial level with respect to an experimental condition rather than averaged activation on single electrodes and time windows, such approaches can be seen as complementary to classical univariate ERP analyses [5]. Classification approaches are less dependent on a priori assumptions (e.g., selection of electrodes or time windows), and naturally simplify the problem of multiple comparisons [6]. In this way, these methods have higher sensitivity by exploiting the interdependence of EEG signals while omitting the information loss due to trial averaging. Moreover, additional analyses allow characterizing the cortical representation of a high number of stimuli, e.g., their dynamics and similarity [5, 7]. Nevertheless, these methods should be considered as a complement to the classical univariate methods, since directional effects cannot be represented. To study the neuronal response to sensory stimuli on single-trial level decoding approaches are often used in a time-resolved manner to investigate the time course of information density in relation to the stimulus. This includes determining the times at which it is possible to determine the nature of a stimulus based on the neural response to it. Determining the dynamics of cortical stimulus processing is thus captured in a data-driven manner, providing a complement to classical ERP analyses. For example, visual object perception [8] or working memory were investigated using decoding approaches [9]. Recently, Vahid et al. [10] identified predictive neurophysiological processes related to N1 and N2 time windows on a single-trial level associated with selective attention using machine learning methods and highlighted the possibility of such methods to validate and form a new hypothesis in a data-driven way. Moreover, López-García et al. [11] successfully applied time-resolved decoding to study selective attention in a flanker paradigm and confirmed interference processing being reflected in the N2 time window on a single-trial level with this approach. Comparing decoding performance across groups could provide new insights into differences in the cortical representation of stimuli or tasks that would not have been possible with conventional EEG analyses. So far, this has been done for different age groups [, 12, 13] and patient groups [14]. The use of machine learning to derive generalizable principles from the complex interaction of time series data could, therefore, add value to the study of lifelong changes in the neural representation of selective attention by confirming hypotheses in a data-driven manner and exploring important implications for its applications [, 15, 16].

Extending a previous analysis by Reuter et al. [4], we aimed to use machine learning to further explore selective attention across the lifespan by decoding the neural representation of inhibitory control using classification in different age groups. We further aimed to explore the classification of age groups to infer age-related changes in selective attention. We hypothesized to classify the type of stimulus and group membership above chance in all groups. We further assumed different decoding trajectories in decoding stimulus types.

## Methods

### Data set

We used data from a total of 222 participants originally collected in three experimental studies each focusing on a different age group. Data sets comprised of EEG data recorded during a modified Flanker task [4]. The full data set includes 92 data sets collected in the setting of the Bremen-Hand-Study@Jacobs [, , 17–19] (Study 1), 81 data sets as part of the re-LOAD project [20, 21] (Study 2) and 49 data sets as part of the CEBRA project [22] (Study 3). The data were first analyzed in a comprehensive manner in Reuter et al. [4] including all 222 participants. All adult participants gave their written informed consent. For children, guardians gave their written informed consent and children agreed to participate. For Study 1 and Study 3 the German Psychological Society and for Study 2 the Ethics Committee of the Faculty of Humanities of the Saarland University, Germany, granted ethical approval. Participants older than 65 scored higher than 27 in the Mini-Mental State Examination (MMSE, [23]) or at least 23 in the Montreal Cognitive Assessment (MoCA, [24, 25]). Participants are separated in the following age categories [4]: children (8 to 10 years), young adults (20 to 29 years), early middle-aged adults (36 to 48 years), late middle-aged adults (55 to 64), old adults < 75 (66 to 75 years), very old adults > 75 (76 to 83 years). We excluded eight participants from further analysis as they had less than 35 correct trials in one of the conditions. Due to poor EEG data quality, we further excluded five participants. Group characteristics included in the final data set are displayed in Table 1.

**Table 1:** Group characteristics

Group	*N* (female)	Age		Number of trials									
				Incongruent					Congruent				
		Mean	Std	*N*	Mean	Std	Min	Max	*N*	Mean	Std	Min	Max
Children	46 (23)	9.32	0.65	3262	70.91	15.30	36	98	3026	65.78	13.27	38	94
Young	39 (34)	22.85	2.50	2554	65.49	24.31	40	109	2476	63.49	22.59	36	109
Early-ma	21 (12)	42.62	3.61	2038	97.05	10.33	79	125	1951	92.90	8.39	75	106
Late-ma	25 (14)	59.04	2.39	2378	95.12	10.65	71	109	2372	94.88	9.01	80	113
Old < 75	40 (36)	71.93	3.04	2355	58.88	19.73	40	111	2263	56.58	14.42	41	92
Very old > 75	38 (30)	78.14	1.94	2632	69.26	21.07	39	105	2506	65.95	21.60	36	101

*Late-ma* late middle-aged, *early-ma* early middle-aged, *std* standard deviation, *min* minimum, *max* maximum

### Experimental procedures

All participants performed a modified version of the Flanker task previously reported in Reuter et al. [17],Winneke et al. [19, 56] and summarized in Reuter et al. [4]. The stimuli consisted of four circles surrounding a target circle in the middle. The target circle was either set to red or green and the task was to press the corresponding button with the index or middle finger of the right hand as fast as possible. The surrounding (flanking) targets were either set to blue (neutral condition) to the same color as the target (congruent condition) or the opposite color, i.e., green target and red flanker and vice versa (incongruent condition).

The experimental procedures were identical between all studies except for trial number and stimulus duration. In Study 1 and Study 3, participants performed 300 trials (approx. 100 trials per condition), whereas in Study 2, they performed 150 trials (approx. 50 trials per condition) in randomized order. Stimuli were presented for 200 ms in Study 1 and Study 3, whereas in Study 2, stimuli were presented for 500 ms. Each trial started with a white fixation cross (300 ms), next a blank screen (200 ms) was presented followed by the presentation of the stimulus and a variable intertrial interval of about 950 ms (i.e., 800 ms to 1100 ms). Participants did a minimum of 20 practice trials and were asked to respond as fast and precisely as possible. Only congruent (no inhibitory control) and incongruent (inhibitory control) conditions as well as correct trials, i.e., trials with a correct response between 100 ms and 1200 ms after stimulus onset, were considered in the following analyses.

### EEG recordings and preprocessing

EEG was recorded with the same 32 Biosemi active electrode system (ActiveTwo, BioSemi, Amsterdam, Netherlands) throughout all studies. Electrodes were placed according to the 1020 system (Jasper, 26). Six additional electrodes recorded vertical and horizontal eye movements as well as mastoid potentials. The sampling rate was set to 2048 Hz and an online band pass filter between 0.16 and 100 Hz was used. Prior to classification we downsampled the data to 256 Hz and filtered between 1 Hz and 40 Hz using the default FIR filter implemented in MNE-Python (version 1.1). Next, we cut the data to segments of 900 ms, i.e., − 100 ms to 800 ms from stimulus onset.

### Machine learning

For classification we relied on a combination of spatial filtering and classification using support vector machines (SVMs) with radial basis function (rbf) kernel to classify the age group and stimulus type. Spatial filtering allows to extract induced spatial patterns, i.e., neuronal responses to external stimuli at single-trial level with not phase locked dynamics, and is thus advantageous compared to the creation of averages across trials [27, 28]. To enhance signal to signal plus noise ratio, i.e., to enhance ERP responses we used the xDAWN algorithm [29]. The xDAWN algorithm was originally developed for P300 evoked potentials in the BCI context and subsequently extended to any type of ERP (see Cecotti and Ries [27] for an overview). Compared to other spatial filtering methods like principal component analysis or independent component analysis xDAWN was shown to be more suitable for the analysis of ERPs aiming at estimating temporal and spatial signatures [30]. Based on the preprocessed EEG segments (cf.2.3), five spatial filters were trained using the training data only (cf.2.4.1). Spatially filtered EEG signals were then classified using a SVM with rbf kernel. Scikit-learn (version 1.1.1), MNE (version 1.1), and imbalance-learn (version 0.10.1) were used to implement the machine-learning pipelines.

#### Classification of age group

We classified group membership on a trial-by-trial basis using all participants data to build a model capable of predicting the associated age group for each trial. We trained and tested our xDAWN + SVM model using stratified tenfold cross validation. We, therefore, randomly split the data 10 times using all trials (N trials per fold: 21538.50 ± 363.91) of 188 participants for training and all trials (N trials per fold: 2981.30 ± 365.50) of 21 participants for testing our model preserving the percentage of samples for each class, i.e., age group. To account for class inequality, we randomly subsampled the training data to the minority class in each fold. As such the same number of trials per age group was present in the training data. In addition to a model using all time samples, we aimed to capture time-resolved decoding performance. That is, we trained and tested repeatedly based on 20 data points with 19 overlapping data points and thus iterated from the beginning to the end of the trial to draw conclusions about decoding performance over time.

#### Classification of stimulus type

Stimulus type classification (congruent/incongruent) was performed within participants, i.e., analogous to the group-level procedure, we trained and tested a xDAWN + SVM model for each participant individually. We divided all trials 10 times randomly in a training set (N trials: 113.61 ± 33.44) and a testing set (N trials: 28.90 ± 8.33) preserving the percentage of samples for each class. We trained and tested the models in a windowing approach using 20 data points with 19 data points overlap to infer decoding performance over time and to be able to compare this between age groups to determine the most discriminative features, i.e., time windows.

## Statistics

We calculated statistics with python using scipy (version 1.8.0) and statsmodels (version 0.13.2). For group classification we report the confusion matrices including precision and recall. In addition, we calculated the accuracy score for each time window. To test if the model of age group classification performed above chance level, we calculated the threshold over which the classification results could be considered significant. We, therefore, relied on the method described in Combrisson and Jerbi [31]. Compared to a permutation approach which randomly shuffles the labels (e.g., 1000 times) with the aim to create a null distribution against which the significance of a model can be tested, this method uses a binomial cumulative distribution to estimate the significance threshold of a classifier. Since permutation approaches are computationally very expensive, especially for large data sets and time-resolved decoding, we relied on this approach due to its computational efficiency and suitability. Due to the large number of trials and our time-resolved decoding approach, it was not feasible to generate a robust null distribution using a high number of group-level permutations. We had a sufficiently large database at the group level, which was more tolerant of deviations from the assumption of a binomial distribution due to cross-validation parameters, such as classifier type and feature space [32]. Besides, comparable results between this approach and a permutation approach were reported [31].

For task classification at an individual level, we reported the area under the receiver operating characteristic curve (AUC) as a mean over all cross-validation folds for each time window. We relied on this metric rather than accuracy as López-García et al. [11] emphasized higher sensitivity of this score compared to accuracy for task classification over time. Since the number of trials within a subject was much smaller than on group levels and for this reason the assumption of a binomial distribution could not be accepted, we used the permutation approach on this level to test the models of stimulus type classification against chance [33]. Therefore, we shuffled the labels 1000 times for each participant creating a null distribution at the group level. From this distribution, we derived the threshold over which the classifier performance could be considered significant at an alpha level of 0.05. We extracted the maximum AUC score and timepoint of maximum AUC score for each participant. In the case of normal distributed data, we conducted one-way ANOVAs to assess the effects of age group on maximum classification performance and timepoint followed by *t* tests for post hoc comparisons. Otherwise, we used nonparametric Kruskal-Wallis test followed by Dunn’s tests for post hoc comparisons. For all tests, the alpha level was set to 0.05 and false discovery rate was used to correct for multiple comparisons [34].

## Results

### Classification of group membership

**Fig. 1:**
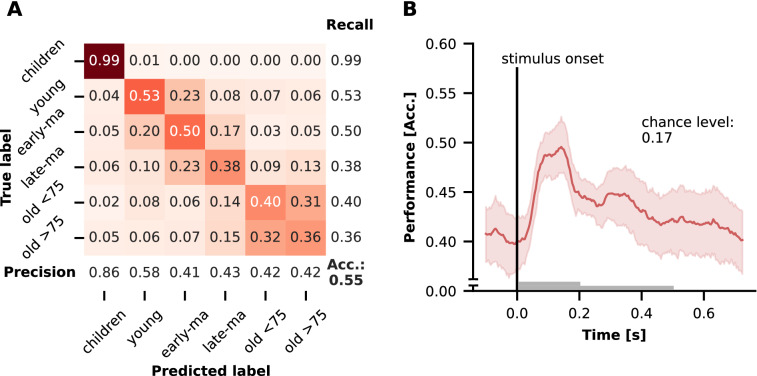
Classification Performance of group membership classification. **A** Confusion matrix including precision, recall for each group as well as the accuracy score (Acc.). **B** Accuracy as a function of time as mean over folds with 95% confidence interval. The gray bars correspond to the stimulus duration. *Late-ma* late middle-aged, *early-ma* early middle-aged, *Acc* accuracy

**Fig. 2:**
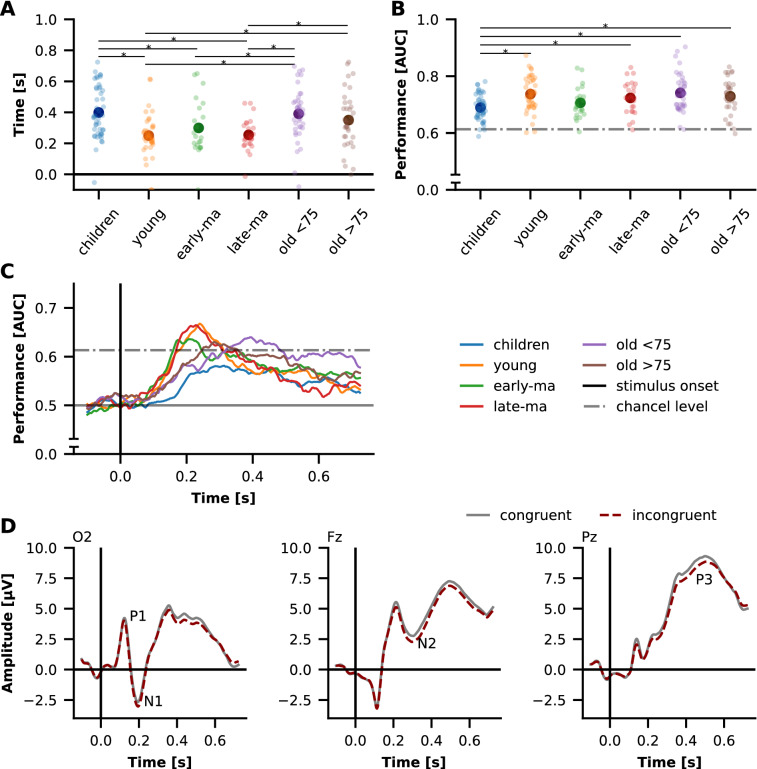
Performance for decoding of stimulus type. **A** Time of maximum decoding performance. **B** Maximum decoding performance. Each point represents one participant, large points represent the group average. *Denotes a significant difference. **C** Group means decoding trajectories. **D** Whole data set ERPs at electrodes O2, Fz, Pz. *AUC* area under the receiver operating characteristic curve,  *late-ma* late middle-aged, *early-ma* early middle-aged

The classification of group membership is presented in Fig. 1. Classification was well above chance level (accuracy = 0.55, chance level: 0.17 at *p* = 0.05). Visualizing misclassifications in a confusion matrix (see Fig. 1A) a clear group structure emerged. While the classification of the children's group worked best, trials in the remaining groups were increasingly classified into adjacent age groups. Here, a larger number of misclassifications can be observed between the younger and early-/late-middle aged adults and a clear cut off to the older adults < 75. Within the two older groups old > 75 and old < 75, however, the highest number of misclassifications can be observed. With respect to time-resolved decoding classification performance was up to 10% higher during the task. The classification performance increased shortly after stimulus onset, with the maximum between 100 ms and 200 ms which is where N1 and P1 are typically reported (see also Fig. 2D). Thereafter, the classification performance decreased until the end of the trial (see Fig. 1B).

### Results of task classification and their differences between groups

Task Classification was possible above chance level for 95.75% of all participants (chance level: 0.61 at *p* = 0.05, see Fig. 2B). Classification failed in three children, two young adults, and one participant in each of the other groups. The AUC score significantly differed between the groups [F(5206) = 4.805, *p* < 0.001]. Post-hoc comparison revealed significant lower scores in children compared to young adults, late middle-aged adults, old adults < 75 and very old adults > 75 (*p* < 0.05).

While the maximum AUC score occurred around 200 ms to 300 ms in the young, early middle-aged, and late middle-aged adults (see Fig. 2A , C), this was later (at approximately 400 ms) in the children and both groups of older adults (old adults < 75 and very old adults > 75). Overall, however, this timepoint was more dispersed among the children and old. The timepoints corresponded to the ERP components N1, N2, and P3 (see Fig. 2D). Kruskal-Wallis test revealed significant group differences for this [H(5) = 35.575, *p* < 0.001]. Children’s AUC scores peaked significantly later than AUC scores of young, early middle-aged, and late middle-aged adults. In addition, the maximum AUC score occurred later in old adults < 75 compared to early middle-aged adults. Further old adults < 75 and very old adults > 75 had later peak AUC scores than the young and the late middle-aged adults (all *p* < 0.05, see Fig. 2A).

## Discussion 

In this study, we aimed to extend previous results [4] on the development of selective attention (as one part of inhibitory control) across the lifespan. We used classification algorithms on EEG data recorded during a flanker task to infer group differences in the dynamics of central processing of selective attention by decoding group membership and the type of stimulus presented at the individual level. Both, decoding of group membership and decoding of stimulus type were significantly above chance. By studying the decoding performance over time, we found that differentiation of groups was performed best early after stimulus onset at around 100 ms to 200 ms. Furthermore, we found different trajectories of decoding the stimulus type between groups. While decoding performance in younger adults to late middle-aged adults was maximal at about 200 ms, this was highly variable in children and older adults. On average, the period around 400 ms was most important for decoding the stimulus type in these groups.

### Classification of group memberships peaked early after stimulus onset

The classification of group membership was above chance level. Precision and recall of the group of children were highest. Misclassified trials in the other groups were mainly assigned to adjacent age groups. Overall, this reflected the age structure of the sample. While the gap between children and the next older group of young adults was quite large, it was smaller for the other groups. However, it should be emphasized that misclassification in the two oldest age groups of old adults < 75 and very old adults > 75 years was quite high in comparison and occurred mainly within these groups. This is certainly partly a reflection of the data structure but could also be an indicator for the high variability of neural activity within these age groups as aging trajectories are highly individual [35]. It should also be noted that there was almost no misclassification between the two oldest groups and children. While ERP markers in the literature on lifetime changes of selective attention are often described as u-shaped [3, 4], our results may highlight that different mechanisms at the beginning and the end of this u-shaped trajectory, i.e., differentiation in children and dedifferentiation in older individuals, are reflected in different brain activation patterns. Furthermore, late middle-aged adults were less likely to be misclassified, although the age gap to the adjacent groups was comparable to that between the two older groups. Late middle-aged adults were also rarely classified as older, which could indicate strong changes in cognitive performance after reaching retirement age (65 years in Germany). We are unable to shed light on this due to a lack of data on participants' retirement. However, a decline in cognitive abilities and cerebral perfusion after retirement is reported in the literature [36–38]. Our results suggest that this is also detectable at the neural level based on EEG recordings which might argue for the diagnostic value of task-related EEG.

Classification performance increased shortly after stimulus onset, peaked at 100 ms and 200 ms and gradually decreased until trial offset. While group differences based on averaged ERP data were described in the components N1, P1, N2, and P3 in previous research [4], we were able to show here that even at the single-trial level group-differentiating information is present. Instead of selected timepoints and electrodes, our analysis further allowed us to determine in a data-driven way at which timepoints the EEG signal was best differentiable between groups. The peak in decoding performance was present at 100 ms to 200 ms in which P1 and N1 ERP components are present (see Fig. 2D) indicating that the early components contribute most to the classification. These ERP components are typically discussed in relation to sensory inhibition and visual attention [39, 40]. Other studies have found P1 and N1 components in children and older adults to be of greater amplitude and latency [41–44]. In the previous study, large amplitude differences were found between age groups in N1 and P1 components, which was interpreted as a reduction in intracortical inhibition in children and older adults, and as increased visual attention in older adults [4]. The high decoding performance found just in this period underlines these previous findings at the level of single-trials. However, maturation processes in childhood and changes in scalp to scull conductivity over the lifespan could have had an influence [45].

In general, classification performance during the task was up to 10% higher than before the onset of the stimulus. This highlights the added value of task-related EEG. Thus, task-related EEG could be used in clinical contexts to predict, for example, the cognitive status of patients. This could be especially of interest in contexts, such as the classification of mild cognitive impairment, where resting state EEG alone could have limited power [46, 47].

### Trajectories of decoding stimulus type differed between group

Peak performance of stimulus type decoding at individual level was above chance level for over 95% of all participants which is in line with previous ERP results finding differences between stimulus types in N2 amplitude and P3 amplitude and latency [3, 4]. There were no group differences in maximum decoding performance between all adult groups. In children, on the other hand, decoding performance was significantly lower. The classifier was less able to distinguish between trials that required inhibitory control and those that did not in this group. At the behavioral level, lower performance in attentional control tasks has been reported in children and it is proposed that the ability to process interfering information develops slowly in children [48]. In fact, this could also be shown in the present data at the behavioral level [4]. Here, especially in children, a large interference effect was shown, i.e., a lower accuracy in the incongruent compared to the congruent condition, which could indicate that inhibitory control has not yet fully been released. Moreover, it was shown from ERP amplitudes of N2 and P3 components that these are less differentiated between stimuli with different attentional demands in older adults but also in children confirming this assumption on the neural level [4, 49]. However, only marginal interaction effects between stimulus type and age group were found based on classical ERP analyses [4]. Using machine learning we go beyond these earlier ERP results, as we found different time curves or trajectories of classification performance between the age groups, characterized by different time windows of maximum decoding performance. Here, we found high variability in children and the two oldest age groups suggesting a high degree of individuality in visual attention in these age groups which may reflect growth in children or deterioration in older adults. For younger to late middle-aged adults, the trajectories were very similar. The highest decoding performance was observed in the time frame around 200 ms, suggesting the importance of this time frame for discriminating between congruent and incongruent conditions. This is consistent with findings on decoding stimulus type in tasks that capture inhibitory control [10, 11]. ERP components N1 and N2 also occur during these periods (see Fig. 2D and Reuter et al. [4]). N1 was shown to be modulated by early visual attentional processes [50] and N2 was discussed as marker of conflict processing in flanker tasks [51]. In contrast, maximum decoding performance in the two oldest age groups was delayed and peaked at approximately 300 ms to 400 ms, suggesting that later timing and components are critical for classification between stimulus categories in these age groups. This difference in the time windows that allow to differentiate between trials with inhibitory control and trials without was not reflected in the previous ERP analyses [4] and could indicate the general slowing of cognitive processes [52].

While decoding or classification approaches have been used in aging research to map neural distinctiveness in the visual [2] or motor system [13, 53] related to dedifferentiation, we show here that changes in the dynamics of stimulus processing can also be identified using machine learning methods. The methods made it possible to map the processing of stimuli at the individual level. On one hand, this could provide additional diagnostic value and be tested in clinical applications. On the other hand, the identified differences between the age groups could have implications for the development of technical systems for the automatic identification of attentional states.

### Methodological considerations 

By combining different data sources, it was possible to access a large data set for our analyses based on machine learning. However, there are small differences between the individual studies due to methodological differences in Study 2. In particular, the presentation length of the stimulus was longer in this study. Although, subjects of Study 2 were included in three, the younger and the two older, age groups and we cannot observe many misclassifications between these age groups in the group classification, we assume that these differences did not have a large impact on the results [4]. Furthermore, the data set of Study 2 consisted of female participants only. For this reason, female participants were overrepresented in the young adult group and the two older groups, but the influence of gender in this data set was already estimated to be small [4]. Last, the number of trials performed was lower in Study 2. To have a database as large as possible as a basis for the machine learning analyses and to reduce selection errors, we decided against rectifying a certain number of trials. Furthermore, the influence of the number of trials can be assumed to be small [10]. Another influencing factor we would like to point out is the influence of different noise levels in the EEG data, which might have influenced the classification and thus the group comparisons. It is unclear and data set specific how large the effect is on the classification performance. Our previous analyses with different preprocessing strategies showed comparable results. Unlike the raw data, we further used xDAWN spatial filtering to maximize signal to signal and noise ratio at the individual level. Finally, we would like to point out that this analysis should be seen as a complement to previous ERP results, since we are examining the information content of stimulus processing to study differences in the cortical representation of inhibitory control across the lifespan but cannot make any statement about the direction of the effects. However, using machine learning methods in this work, we were free of assumptions regarding the localization of effects in space (electrodes) and time (time windows) and were able to achieve a higher sensitivity to stimulus effects across the lifespan. This approach can be attributed to decoding or MVPA approaches and could be used in studies with higher number of stimuli as starting point for further analyses to study the representational structure of stimuli as it is done in the representation similarity analysis (RSA) framework [7]. Thereby, the performance of classifiers serves as a measure for the difference between two brain activity patterns. Cross validated metrics, as used in this study, are considered advantageous [54].

## Conclusion

In summary, we were able to extend previous results using machine learning techniques to detect age and task differences in cognitive processing on a single-trial level. This is especially crucial for a step behind classical ERP components and a more direct link between behavior and neural dynamics [55]. The data-driven approach used in this research particularly highlights early attentional processes in the classification of age groups and suggests the benefit of task-related EEG data in the classification of different age groups, which could be used in clinical contexts. With respect to information processing in selective attention our analyses could confirm the relevance of time windows corresponding to N1 and N2 components reported in ERP studies. Furthermore, the time windows relevant for inhibitory control differed between groups, i.e., later time windows were relevant in older adults, suggesting that different processes are important for selective attention at different ages. Overall, we showed that using machine learning compared to a priori selected electrodes and timepoints, we were able to obtain assumption-free insights into differences in inhibitory control over the lifespan. Machine learning thus represents an extension to classical methods that can be used to test existing theories but also to extend them.

## Data Availability

The data sets used and/or analyzed during the current study are available from the corresponding author on reasonable request. Python source code is available: ∙ Project name: ML_selective_attention. ∙ Project home page https://github.com/christiangoelz/ML_selective_attention. ∙ Archived version: https://doi.org/10.5281/zenodo.7551173. ∙ Operating system(s): Platform independent. ∙ Programming language: Python. ∙ Other requirements: Python 3.7 or higher, for packages see file ‘requirements.txt’.

## Abbreviations

BCI: Brain–computer interface
EEG: Electroencephalography
ERP: Event-related potential
MMSE: Mini-mental state examination
MoCA: Montreal Cognitive Assessment
MVPA: Multivariate pattern analysis
rbf: Radial basis function
RSA: Representation similarity analysis
SVM: Support vector machine
